# Metabolism, HDACs, and HDAC Inhibitors: A Systems Biology Perspective

**DOI:** 10.3390/metabo11110792

**Published:** 2021-11-20

**Authors:** Jacob King, Maya Patel, Sriram Chandrasekaran

**Affiliations:** 1 Department of Biomedical Engineering, University of Michigan, Ann Arbor, MI 48109, USA; kingjaco@umich.edu (J.K.); mayanp@umich.edu (M.P.); 2 Department of Computational Medicine and Bioinformatics, University of Michigan, Ann Arbor, MI 48109, USA; 3 Program in Chemical Biology, University of Michigan, Ann Arbor, MI 48109, USA; 4 Center for Bioinformatics and Computational Medicine, University of Michigan, Ann Arbor, MI 48109, USA; 5 Rogel Cancer Center, University of Michigan Medical School, Ann Arbor, MI 48109, USA

**Keywords:** epigenome, gene regulation, histone acetylation, histone deacetylases, proteomics, transcriptomics, metabolomics

## Abstract

Histone deacetylases (HDACs) are epigenetic enzymes that play a central role in gene regulation and are sensitive to the metabolic state of the cell. The cross talk between metabolism and histone acetylation impacts numerous biological processes including development and immune function. HDAC inhibitors are being explored for treating cancers, viral infections, inflammation, neurodegenerative diseases, and metabolic disorders. However, how HDAC inhibitors impact cellular metabolism and how metabolism influences their potency is unclear. Discussed herein are recent applications and future potential of systems biology methods such as high throughput drug screens, cancer cell line profiling, single cell sequencing, proteomics, metabolomics, and computational modeling to uncover the interplay between metabolism, HDACs, and HDAC inhibitors. The synthesis of new systems technologies can ultimately help identify epigenomic and metabolic biomarkers for patient stratification and the design of effective therapeutics.

## 1. Introduction

Histone acetylation is a reversible epigenetic modification regulated by two opposing enzymes, histone acetyltransferases (HATs) and histone deacetylases (HDACs), which transfer acetyl moieties to and from lysine residues of target proteins, respectively. This type of epigenetic regulation plays a role in a number of pathological conditions, including hypercholesterolemia, obesity, neurodegenerative disorders, cancer, and cardiovascular diseases [1,2]. For example, HDACs promote cancer cell growth by repressing the expression of tumor suppressor genes such as p21 and p53 [1]. Interestingly, recent studies suggest that HDACs may play other roles outside of transcriptional regulation, such as regulating metabolism [3,4]. Altered metabolic processes are also present in the above-mentioned disorders. For instance, cancer cells often display increased glycolysis, decreased oxidative phosphorylation, and increased synthesis of metabolites involved in cell proliferation [4,5]. Of interest, several metabolites can act as activators or inhibitors of HDACs. Thus, understanding the relationship between HDACs and metabolic activity can lead to the discovery of agents and targets for treatments of these conditions. Here, we provide an overview of the crosstalk between HDACs and metabolism with an emphasis on metabolites that act as histone deacetylase inhibitors (HDACIs).

HDACs catalyze the deacetylation of lysine residues in both histone and nonhistone proteins. Deacetylation of histones limits the expression of target genes. Acetylation of histone tails by HATs removes the positive charge from the N-terminal, disrupting the electrostatic interaction between histones and negatively charged DNA. This creates an “open” chromatin structure and increases accessibility of transcription factors, thus promoting gene expression. Opposingly, deacetylated histones maintain their positive charge and remain tightly bound to negatively charged DNA, thus limiting the accessibility of transcription factors, and suppressing gene expression [1,6,7]. HDACs are also responsible for the deacetylation of nonhistone proteins, such as RUNX3 (a tumor suppressor) and HSP90 (a molecular chaperone) [6]. While deacetylation of histones has transcriptional effects, deacetylation of non-histones can affect protein stability, interactions, and signaling [7]. 

In humans, 18 known HDACs are classified into four categories that differ in structure, localization, and cofactor requirement. Class I HDACs are usually located in the nucleus, while class II HDACs are found in the nucleus and cytoplasm [8,9]. HDAC11 is the only class IV HDAC. It is active in the nucleus, shares a similar structure to class I/II HDACs, and regulates the stability of CDT1, a DNA replication factor [8]. Class I, II, and IV HDACs are all zinc-dependent. On the other hand, class III HDACs, known as Sirtuins, are NAD^+^ dependent. The primary structure of Sirtuins is similar to that of the yeast Sir2 protein, and they can be active in the nucleus, mitochondria, or cytoplasm (depending on the specific Sirtuin) [8]. 

Many small molecules have been identified as histone deacetylase inhibitors (HDACIs). These molecules fall into several categories, including hydroxamates (e.g., SAHA/Vorinostat), cyclic tetrapeptides (e.g., FK228/Romidepsin), aliphatic acids (e.g., valproate, butyrate), and benzamides (e.g., MS-275/Entinostat) [10]. Inhibitors of class I, II, and IV HDACs function via a zinc-binding group that directly interacts with the zinc ion in the HDAC catalytic site [11]. Studies regarding HDACI treatment of tumors show transcriptional de-repression of tumor suppressor genes, leading to increased cell cycle arrest and decreased metastasis and angiogenesis. Specifically, SAHA (Vorinostat), FK228 (Romidepsin), LBH-589 (Panobinostat), and PXD101 (Belinostat) are FDA-approved drugs that treat specific cancer types, mainly T-cell lymphomas [10]. HDACIs Ricolinostat (ACY-1215) and citarinostat (ACY-241) are currently in clinical trials [12]. Current trials are also examining the use of HDACIs on solid tumors, as well [13]. Although the transcriptional impact of HDACIs has been well-studied, new research is revealing ways HDACIs impact cell growth and phenotype via metabolic mechanisms [13]. 

## 2. Relationship between HDACs, HDACIs, and Metabolism

Recently, HDACs have been shown to regulate proteins other than histones, such as enzymes involved in metabolic pathways. There are several examples of HDACs that affect metabolic pathways. Knutson et al. and Feng et al. studied the effects of HDAC3 disruption and deletion in the liver, which plays a central role in metabolism and detoxification in the body [14,15]. Results of the Knutson et al. study showed that mice without HDAC3 were smaller than control mice [14]. Further histological analysis via mass spectrometry revealed significantly decreased concentrations of glycogen and increased concentrations of lipids (specifically triglycerides and cholesterol) in hepatocytes of mice lacking HDAC3 [14]. Feng et al. also reported hepatic steatosis, a condition marked by abnormally high retention of lipids in the liver, in mice lacking HDAC3 [15]. The mechanism of this phenomena was uncovered by Sun et al., who found that HDAC3 recruits metabolites such as acyl-CoAs away from lipid synthesis and towards gluconeogenesis to increase glucose production in the liver [16]. Taken together, the results demonstrate that HDAC3 plays a significant role in liver metabolism.

Yang et al. demonstrated another example of how metabolism is affected by HDACs, specifically related to hepatocellular carcinoma (HCC) [17]. In many cancers, including HCC, increased aerobic glycolysis leads to enhanced tumor growth, as described by the Warburg effect [18]. The gluconeogenesis pathway suppresses aerobic glycolysis, and hence, inhibiting gluconeogenesis can also contribute to cancer cell growth. Yang et al. found high concentrations of HDAC1 and HDAC2 in HCC tissues [17]. HDAC1 and HDAC2 suppress the expression of Fructose-1,6-bisphosphate (FBP1), the rate limiting enzyme in the gluconeogenesis pathway, by deacetylating histone H3K27 in the *FBP1* enhancer [17]. This downregulates gluconeogenesis, thus promoting aerobic glycolysis and cancer growth. Upon knockdown of HDAC1 and HDAC2, HCC cell lines showed increased FBP1 expression and decreased cell growth [17]. Understanding how HDACs impact cancers through metabolism can provide novel targets for potential treatments.

**Figure 1:**
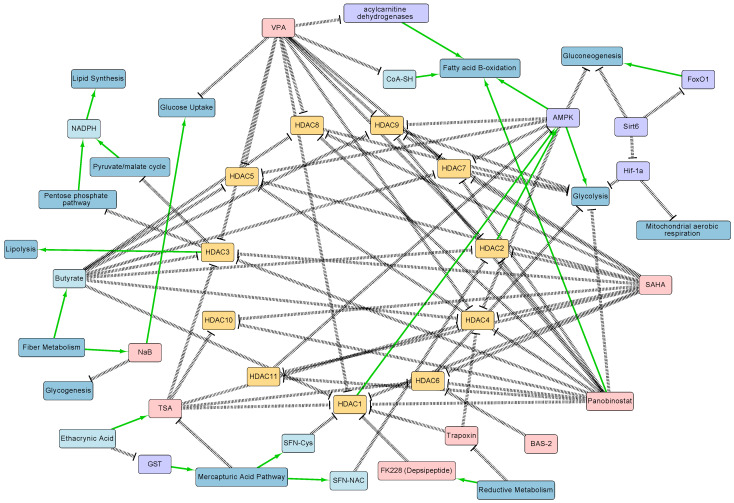
HDAC–metabolic interaction network diagram generated from literature curation. Salient regulatory interactions between HDACs 1-11, metabolites, and metabolic pathways are shown. Note that this diagram is not comprehensive and does not show all interactions between these processes. HDACs are represented in yellow, HDAC inhibitors in red, metabolites in light blue, metabolic pathways in dark blue, and proteins in purple. Positive regulatory arrows are depicted in green and negative regulatory arrows in black. The network map was created in Cytoscape. The figure highlights the complexity of the interactions between HDACs and metabolism, and highlights the need for systems biology approaches to study their interplay.

Since many HDACs regulate metabolism, inhibition of HDAC activity through HDACIs impact various metabolic processes (Figure 1). Amoedo et al. treated lung cancer H460 cells with known HDACIs, sodium butyrate (NaB), and trichostatin A (TSA) [19]. NaB is the sodium salt of butyrate, a short-chain fatty acid. TSA is a natural derivative of dienhydroxamic acid. HDAC inhibition occurs when the deprotonated carboxyl group of NaB or the hydroxamic acid moiety of TSA bind to the zinc atom of Class I HDAC active sites. NaB-treated H460 cells had high rates of oxygen consumption coupled to ATP synthesis, and increased activation of the pentose phosphate pathway (PPP) [19]. Both HDACIs also promoted mitochondria bound hexokinases activity, thus increasing glycolysis [19]. Overall, the study showed that HDACIs NaB and TSA increased aerobic and mitochondrial metabolism.

A study by Wardell et al. evaluated the effects of HDACIs valproate (VPA), a short-chain fatty acid, and suberoylanilide hydroxamic acid (SAHA), a synthetic hydroxamic acid derivative, on metabolism in multiple myeloma [3]. These HDACIs had several metabolic effects in the cells: decreased acetyl-CoA levels, downregulated glucose transporter type 1 (GLUT1), and inhibited hexokinase 1 (HXK1) activity [3]. The latter two effects contribute to a decrease in glucose uptake and glycolysis, on which cancer cells rely heavily for energy [18]. For energy, the cancer cells must then turn to fatty acid or amino acid catabolism. Due to the lower acetyl-CoA levels, however, fatty acid β-oxidation cannot occur. The cancer cells are thus forced to utilize amino acid oxidation for energy, which leads to apoptosis [3]. These results demonstrate the mechanism by which therapeutic anticancer HDACIs affect metabolism and show the potential of these agents as drugs for cancers that rely on GLUT1 and HXK1 for catabolism. 

In addition to changes in glucose uptake and glycolysis, HDACIs can alter the levels of glutamine, a central metabolite involved in multiple pathways. Deshmukh et al. described how HDAC inhibition leads to a decrease in plasma glutamine levels, which helps to reverse tumor growth [20]. This discovery has led to new research into treating cancer based on glutamine transporters, aimed at blocking SLC1A5 and SLC1A7, thus decreasing glutamine transport and reducing tumor growth. Inhibition of glutamine transport and synthesis, potentially in combination with HDAC inhibition, may represent the next wave of cancer therapeutics. 

While we have so far highlighted the impact of HDACs and HDACIs on metabolism, several metabolites have also been shown to play a role in the regulation of HDAC and HDACI activity. For instance, class III HDACs, known as Sirtuins, are NAD+ dependent [21,22]. NAD^+^ is a redox cofactor for catabolic pathways including glycolysis and fatty acid β-oxidation. There is a clear relationship between NAD^+^ levels and Sirtuin activity. Disturbance of the many metabolic pathways involving NAD^+^ can alter the availability of NAD^+^ for Sirtuin function. For instance, starvation creates an energy restricted metabolic state in the cell and increases the ratio of NAD^+^ to NADH. This increased NAD^+^ concentration allows Sirtuins to increase their activity, potentially leading to a longer lifespan in various model organisms [22]. 

Coenzyme A (CoA) and its derivatives have also been shown to influence HDAC activity [23,24]. In vitro studies and kinetic analysis by Vogelaur et al. showed that several metabolites, specifically acetyl-CoA, butyryl-CoA, HMG-CoA, and malonyl-CoA, are allosteric activators of HDAC1 and HDAC2 [23]. These CoA derivatives speed up the reaction rates of HDACs, increasing their activity by 1.5 to 3-fold [25]. The CoA molecules include intermediates of carbohydrate or amino acid catabolism and precursors to fatty acid or sterol anabolism. These metabolites contain a phosphorylated adenosine on the ribose moiety. NADP, which provides reducing power for anabolic metabolism, also contains a phosphorylated ribose moiety. These results suggest that the phosphorylated ribose group that is found in several metabolites may serve a role in their interactions with HDAC1 and HDAC2. In all, the results of these studies and others provide evidence that several metabolites regulate HDAC function.

Metabolism also plays a major role in modulating the activity of HDAC inhibitors. For instance, trapoxin (TPX) is a microbial tetrapeptide that can induce apoptosis and cell differentiation [26]. Trapoxin is also an irreversible inhibitor of HDAC1 and HDAC4. Furumai et al. and Kijima et al. revealed that the functional unit of TPX is an epoxyketone [26,27]. The epoxyketone is capable of performing alkylation, thus allowing the HDACI to covalently bind to the HDAC target. Further studies revealed that TPX lost its inhibitory ability upon chemical reduction of the epoxide groups to alcohols. Because of this, anabolism, which is reductive, can potentially repress the activity of TPX [2,27].

On the other hand, metabolism can also increase activity of certain HDACIs. One example of this is depsipeptide anabolism. The reduction of the disulfide bond in protein FK228, or depsipeptide, creates an active HDACI [2]. The resulting compound, pharmaceutically known as Romidepsin, can fit in the HDAC pocket, has an inhibitory effect, and is an effective anticancer agent in leukemias and lymphomas [28]. Thus, reductive metabolism of depsipeptide increases inhibition of HDACs. 

Dietary metabolism can also create HDACIs. For instance, fiber is ingested and fermented in the gastrointestinal system into short-chain fatty acids, which can have inhibitory effects on HDACs [1]. A common example of this is butyrate, a competitive HDACI produced via this metabolism of dietary fiber [1]. Other examples include 4-phenylbutyrate and tributyrin [29]. Each of these short-chain fatty acid molecules induce histone acetylation because of their HDACI activity. In all, several examples demonstrate that metabolism can inactivate, activate, or create HDACIs. Thus, the rate and type of metabolism taking place can have major effects on HDACI activity. A summary of the specificity, structure, and metabolic relationship of HDACIs mentioned in this section is displayed in Table 1.

Understanding the relationship between HDACs and metabolism can reveal information about the underlying mechanisms involved in numerous diseases. In addition, metabolites that serve as HDACIs can be applied to drug discovery. For instance, specific metabolites can be used in cancer therapy to inhibit HDACs that repress tumor suppressor genes [1]. Furthermore, research on non-transcriptional HDAC mechanisms, such as those involved in the regulation of metabolic pathways, can provide metabolic or signaling targets for potential combination therapies [3]. However, HDACs regulate numerous proteins that directly or indirectly influence the levels of metabolites, which in turn can feedback and influence numerous HDACs. Therefore, the complexity of the metabolic–HDAC interaction network (Figure 1) in a given cell makes it challenging to analyze their interactions, necessitating the use of systems biology technologies.

## 3. Emerging Technologies to Study Interactions between HDACs and Metabolism

### 3.1. Epigenomics

The epigenome comprises any reversible genetic changes that do not alter the genetic sequence itself but can alter the way genes are expressed. There are four main epigenetic changes that block the accessibility of transcription factors to their binding sites: chromatin structure changes, nucleosome repositioning, DNA methylation, and histone modification. These changes are dynamic and reversible. Since some cells within a tissue or tumor may have a specific epigenetic change, and other cells may not, it is important to employ methods that allow for the analysis of epigenetic variation at the single-cell level. Such methods are being newly developed for epigenomic studies.

There are many methods used to study epigenomes. Bisulfite sequencing (BS-seq) is used to analyze DNA methylation of cytosine residues located in CpG islands. Unmethylated cytosine residues react with bisulfite to form uracil, while methylated residues will not. To apply this method on the single-cell scale, reduced representation bisulfite sequencing (scRRBS) was performed by enriching CpG-dense regions [35,36]. However, scRRBS is not ideal as this method has poor coverage of the epigenome, leaving out some important regulatory regions. To combat this, the post-bisulfite adapter-tagging (PBAT) approach incorporates adapter tagging to prevent the loss of fragments of DNA [35]. One relevant study used BS-seq to describe how trichostatin A, an HDACI, inhibited MMP-9-dependent H3NT proteolysis and likely targets HDAC’s ability to remove acetyl groups from H3K27ac [37].

**Figure 2:**
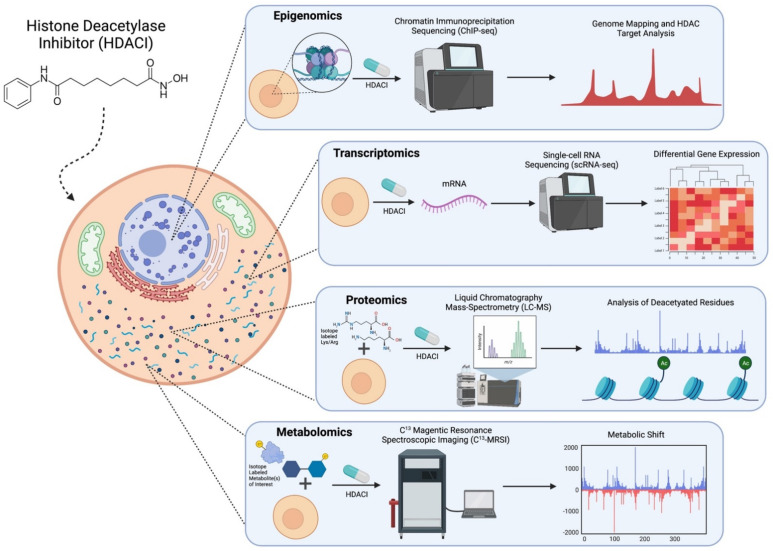
Diagram of emerging technologies such as transcriptomics, proteomics, metabolomics, and epigenomics to study HDACs, HDAC inhibitors, and their interactions with metabolism. Created with BioRender.com (access on 13 October 2021).

By far the most common tool to study the epigenome, especially regarding HDACs and HDACIs, is Chromatin immunoprecipitation sequencing (ChIP-seq) (Table 2). This method involves isolation following the generation of random chromatin fragments via sonication and then purification via an antibody selective for DNA-binding proteins (Figure 2). To apply this to the single-cell level, the cells undergo micrococcal nuclease (MNase) digestion before immunoprecipitation. This reduces background noise and allows thousands of cells to be processed in parallel [35]. ChIP-seq measures general protein binding to DNA, so it can assess histone modifications and chromatin remodeling. This is incredibly useful because researchers can discover where exactly HDACs are located on the genome and thus what genes are regulated by them. Hanian et al. developed their own ChIP-seq method which uniquely utilized a photoreactive HDACI probe and verified the results by measuring the expression of HDACI regulated genes. Their results indicate that HDACIs only target HDACs that are attached to the gene promoter and regulator regions, as opposed to the gene boxes. However, ChIP-seq’s utility is largely seen in multi-omics studies which combine gene expression (RNA-seq) with ChIP-seq data. Rafehi et al. combined both transcriptomic and epigenomic methods by integrating microarray expression data of 33 HDACIs with ChIP-Seq datasets of EP300 target genes. These genes encode transcription factors that are key regulators of immunity, lipid metabolism, and insulin receptor signaling mechanisms in diabetes. Their multi-omics analysis identified the suppression of EP300 genes in diabetic patients following HDACI treatment [38].

### 3.2. Transcriptomics

Transcriptomics studies quantify transcriptional products (mRNA, ncRNA), determine the transcriptional structure of genes (splicing patterns, starting sites), and quantify expression level changes under various conditions [39]. Several researchers have used transcriptomics methods to study the relationship between HDACs and metabolism. Microarrays allow researchers to assay thousands of transcripts simultaneously, but this technology has several drawbacks, including dependence on already known genomes, high background noise, and lower resolution than other methods [39,40]. RNA sequencing (RNA-seq) is the most used transcriptomics approach over the past few decades. This method has very high resolution (single base-pair) relatively inexpensive and offers a large detection range spanning over ~6 orders of magnitude [39,40]. Rafehi et al. analyzed the effects of TSA and SAHA (common HDACIs) on vascular chromatin to uncover novel HDACI functions [38]. RNA-seq revealed that the expression of genes involved in trafficking, neuronal systems, lipid metabolism, and signaling were all increased with increased promoter histone acetylation [38], while genes in RNA processing, DNA repair, nucleotide metabolism, and the cell cycle were all suppressed with reduced promoter acetylation [38]. RNA-seq is a powerful tool to study HDAC inhibitors’ inherent epigenetic regulation by assessing differentially expressed genes. However, traditional RNA-seq will pool many cell types within a tissue together, losing some of the novel cell-types in the mix. 

Single cell sequencing methods have the capability to detect cellular heterogeneity in transcriptomics, genomics, and epigenomics. Fluorescence-activated cell sorting (FACS) is used in many single-cell approaches to isolate the single cells and label them based on specific characteristics [35]. Single cell methods yield specific information about biomarkers of cells of interest (e.g., tumor cells). Single-cell RNA sequencing (scRNA-seq) is a powerful tool that can be used to study the gene expression of these rare cell types (Figure 2). Despite its powerful capabilities, quality control of scRNA-seq samples, differentiating biological from technological noise, and analysis of these high-dimensional data are still significant challenges in this field (Table 2) [41]. Srivatsan et al. developed a novel scRNA-seq method called sci-Plex RNA-seq or sci-RNA-seq which utilizes nuclear hashing to quantify gene expression across thousands of independent cells, while still maintaining the single cell resolution [42]. The study discovered that on a single cell level, HDAC inhibition leads to an upregulation of genes involved in acetyl-CoA, indicating that HDACI-treated cells are engaged in an acetyl-CoA-deprived state [42]. The low levels of acetyl-CoA were likely caused by the sequestering of acetate in acetylated lysine residues, which were increased in response to HDACI treatment [42]. Furthermore, the researchers observed up-regulation of genes involved in citrate homeostasis, transport, and mitochondrial citrate production [42]. Transcriptomic analysis is a robust methodology for studying the metabolic changes induced by HDAC inhibition and can be used to assess differential gene expression in both bulk tissues and single cells. However, expression of mRNA may not necessarily be indicative of translation to protein. 

### 3.3. Proteomics

Recent studies have found a correlation coefficient of only ~0.5 between mRNA and protein levels [43]. This is largely due to three factors. First, mRNA is not always translated into proteins. Second, the transcriptome is less dynamic when compared to the proteome, which changes depending on cell type and time [43,44]. Finally, genomics and transcriptomics do not account for post-translational modifications [45]. Proteomics allows for the analysis of protein dynamics and interactions. Proteomic studies often combine a protein assay, chromatography method, and mass spectrometry (MS) to study protein activity. The proteins of interest are separated by chromatography and analyzed using mass spectrometry. While these methods can be highly specific, mass spectrometry machines can introduce higher operational and maintenance cost into the research (Table 2).

These techniques have allowed researchers to determine post-translational modifications of proteins, specific protein responses to various environmental conditions, drug treatments, and signaling effects. For instance, Schölz et al. used quantitative nanoflow liquid chromatography–mass spectrometry (LC-MS) to discover specific acetylation sites of 19 different HDACIs in HeLa cells [46] (Figure 2). Arginine and Lysine residues were isotopically labeled prior to HDACI treatment. This is known as Stable Isotope Labeling by Amino Acids in Cell culture (SILAC), which is used to label specific amino acids so they can later be detected and measured by MS. After the treatment of HeLa cells with the HDACIs, proteins were purified, and acetylated proteins were enriched using anti-acetyllysine antibodies. These antibodies are specific to the change in interest (lysine acetylation), allowing for the detection of proteins with this modification. The samples were then loaded onto nanoflow liquid chromatography columns, eluted, and ionized using electrospray. Nanoflow liquid chromatography separates samples using slow flow rates and improves peptide ionization, allowing for extremely high sensitivity. Finally, mass spectrometry recognizes and quantifies acetylation sites via the amino acid isotope labeling. Schölz et al. utilized the high sensitivity of nanoflow liquid chromatography MS, which allowed for the identification of specific acetylation sites for 19 lysine deacetylase inhibitors [46]. By analyzing the MS acetylation profiles after HDACI treatment, the researchers discovered that bufexamac, when treated in lower doses, specifically inhibit HDAC6, and at high concentrations, chelate cellular iron, which results in the hypoxia seen in bufexamac treatment [46].

Bryson et al. also used LC-MS proteomics and SILAC to evaluate the specific lysine acetylation sites generated in response to HDACI treatment [47]. The results of this study showed acetylation sites on non-histone proteins, suggesting that HDACIs have additional non-transcriptional mechanisms of action. A connection between post-translational modifications lysine acetylation and tyrosine phosphorylation was also found, suggesting that HDACIs may impact both acetylation and phosphorylation via signaling networks. These methods can be used in studying HDAC functions and mechanisms.

Biotinylation of lysine is another way to uncover HDAC functions and targets. The process involves first, acetylating all available lysine residues. Then, the proteins are treated with HDACs, which will deacetylate certain residues. Biotinylation reactions are carried out, in which biotin binds to deacetylated lysines. Streptavidin-coated beads purify the samples and separate proteins labeled with biotin. MS is implemented to identify the biotinylated residues, which represent the specific targets of the HDACs used. This in vitro technique has been useful in showing HDAC specificity and function. For instance, Bheda et al. used this technique to identify H3K79 as a target of Sir2 [48].

Finally, a high-throughput method combining reduced-representation phosphoproteomic assay (P100) and a global chromatin profiling (GCP) assay can reveal the signaling and chromatin changes in response to specific drugs. P100, first introduced by Abelin et al., uses isotopic labels, immobilized metal affinity chromatography, and MS to identify 100 phosphopeptides and their response to various drug treatments [49]. GCP identifies post-translational modifications to histones that are involved in epigenetics, as shown by Creech et al. [50]. While P100 can identify 100 phosphopeptides, GCP can analyze the post-translational modifications of ~60 histone related peptides. By combining P100 and GCP technologies, Litichevsky et al. evaluated the effects of HDACIs and other drugs in six cell lines including embryonic stem cells (ESC) and neuronal precursor cells (NPC) [51]. By analyzing the response to various drugs, they found that diverse phosphosignaling states corresponded to a smaller number of chromatin states in a cell type-specific manner. The researchers discovered that myeloma cell lines with t4:14 translocation developed a unique profile of post-translational modified histone proteins compared to other cell lines after HDACI treatment [51]. This correlated with the observation that t4:14 translocated lines are likely to have higher sensitivity to HDACIs [51]. Both P100 and GCP can be utilized to study post-translational modifications and thus, when combined with HDACI treatment, can be used to explain cellular response and sensitivity to HDAC inhibition.

LC-MS, lysine biotinylation, P100, and GCP are just a few proteomic methods that reveal important details about HDAC activity and specificity. By revealing post-translational modifications and dynamic effects of HDACs and HDACIs, several studies have suggested additional HDAC mechanisms involved in phosphorylation signaling networks. LC-MS and biotinylation of lysine have allowed researchers to determine the specific number, ratio, and location of HDAC and HDACI target sites. Determining post-translational modifications can provide key insights into HDAC–metabolite interactions, further describing how metabolic shifts occur in response to HDAC inhibition.

### 3.4. Metabolomics

The main approach to study metabolomics is MS. MS is often preferred over other metabolomic technologies because of its higher sensitivity, which allows for the analysis of metabolites in low concentrations; MS can detect pM–attomolar concentrations. MS utilizes an ionization source (most commonly electrospray ionization), mass analyzer, and ion detector to measure the mass–charge ratio. This technique is usually paired with a separation method such as liquid chromatography (LC), gas chromatography (GC), capillary electrophoresis (CE), or supercritical fluid chromatography (SFC). LC-MS, GC-MS, CE-MS, and SFC-MS are approaches that are used to quantify amounts of metabolites in different cells and tissues. Alcarraz-Vizán et al. employed MS to quantify changes in metabolite levels in response to HDACIs using a tracer-based metabolomics approach, which utilizes ^13^C to trace specifically labeled metabolites [52]. Their results indicated that HDAC inhibitors (trichostatin A and butyrate) induce a common metabolic profile in HT29 cells. In contrast, acetyl-CoA precursors induce a distinct metabolic profile compared to butyrate, which is both an HDACI and an acetyl-CoA precursor [52].

Another approach to study metabolism on a cell or tissue level is to use nuclear magnetic resonance (NMR). NMR is based on the specific frequencies at which different nuclei resonate when placed in a strong magnetic field. Data from NMR provide specific information about the chemical properties and structure of the tested compounds. While NMR may not have the resolution or selectivity of other methods, it is both cost- and time-effective, allowing large studies to be undertaken in a relatively short amount of time. Cuperlovic-Culf et al. utilized NMR to study the metabolic changes undergone when five HDACIs were administered [25]. Their results illustrated that different glioblastoma cell lines respond independently, in terms of metabolic shift and cell-survival, to the same HDACIs, while also defining a general increase in Krebs cycle intermediates and proline in the HDACI treated groups [53]. 

Another related approach to NMR and MS is the technique of ^13^C Magnetic Resonance Spectroscopic Imaging (^13^C-MRSI), which can be used to assess the metabolic trends of specific reactions or pathways (Table 2) [54]. This procedure follows a typical tracer-based metabolomics approach that uses ^13^C to label metabolites, but instead of MS, Magnetic Resonance Spectroscopic Imaging is used to assess any metabolic shifts and quantify these changes (Figure 2). This process typically has low resolution, but recent advancements in liquid-state polarization of ^13^C substrates have allowed over a 50,000-fold increase in resolution. Radoul et al. utilized ^13^C-MRSI to study the metabolic effects of HDACIs on lactate metabolism [55]. The researchers decided on MRSI because their goal was to determine if tools used for initial diagnosis, such as MRSI, might be capable of being used in the assessment for treatment. Their results indicate that tumor cells respond to HDACIs with a decrease in lactate production. As explained by the Warburg Effect, tumors have increased rates of glucose uptake and aerobic glycolysis, leading to increased lactate concentrations. Even in the presence of oxygen and functioning mitochondria, tumors favor fermentation of glucose to lactate because rapid ATP generation is favorable for tissue proliferation [5]. Because of this, Radoul et al. propose that the use of MRSI in measuring lactate production can help determine if a tumor is responding to HDACI therapy [55]. ^13^C-MRSI narrows the focus of a metabolic study to just a few metabolites of interest and how these metabolites behave in response to a treatment. These different technologies are used to fulfill distinct tasks in quantifying metabolic shifts. MS is by far the most common and allows for the most precise measurements of metabolites. NMR represents a faster and cheaper alternative that can still provide sufficient results and has been especially useful in the assessment of large studies where time and money constraints are exacerbated. Finally, ^13^C-MRSI has the potential capability of integrating tissue imaging and metabolomics.

### 3.5. High Throughput Cell Line Screening

Cell line screening is another widely emerging methodology that has yet to be fully taken advantage of in studying HDACIs. High-throughput screening methods can be utilized to screen thousands of cells and drugs, with varying types. Through computational and machine learning methods, these high-throughput screens have been able to combine multi-omics data types that have otherwise been difficult to link traditionally. Notable high-throughput cell screening projects that have led to the generation of large datasets include the Cancer Cell Line Encyclopedia (CCLE) and the Cancer Therapeutics Response Portal (CTRP) studies. The CCLE database contains gene expression, metabolite levels, epigenomes, genome sequence, and genomic annotations of 947 cancer cell lines [56]. The CTRP contains the drug effectiveness values of 481 drugs, 21 of which are HDACIs, across 860 CCLE cancer cell lines. These large datasets allow researchers to skip the data generation step and begin right with the analysis. Furthermore, the large breadth these datasets contain allows researchers studying different cancer phenomena to utilize the same dataset. Regarding HDACIs, these two datasets could be used together to study the effects of HDACIs on different cancer lines and thus provide insights into why some cancer cell lines are more susceptible to HDACIs than others [37]. 

Other promising high-throughput screening methods that have yet to be fully utilized in studying HDAC inhibitors are PRISM and other systematic variability profiling methods. PRISM barcodes individual cancer cell lines with a 24-nucleotide sequence, which then can be amplified and analyzed after the assay of choice [57]. Using PRISM, the researchers screened 8400 potential drug compounds across 102 cancer cell lines and identified a novel aurora kinase inhibitor BRD-7880 [57]. PRISM has the potential to further increase the throughput of such screening studies which have yet to be fully utilized when studying HDACs and HDACIs. PRISM has been shown to generate the same results of cell killing as more conventional screening methods, while increasing cell throughput and has already been utilized to identify novel potent aurora kinase inhibitors for cancer treatment. Similarly, Corsello et al. employed PRISM to identify novel and repurposed compounds with anti-cancer effects and characterized their targets [58]. PRISM and other high-throughput methods, especially when combined with novel computational and machine learning methods, will be the clear next step in studying HDAC inhibitors, because this will allow researchers to combine multi-omics data and uncover features predictive of HDACI efficacy. For example, the PRISM study revealed that the sensitivity of cancer cell lines to many compounds can be predicted from the genomic features of the cell lines. This can enable personalized treatments of HDACIs and other drugs based on genome sequence.

### 3.6. Genome-Scale Metabolic Modeling

Genome-scale metabolic modeling represents another emerging methodology for the future of studying HDAC inhibitors. Genome-scale metabolic models (GEMs) computationally describe the entire set of gene–protein–metabolite relationships in an organism [59]. GEMs can be adjusted to simulate different metabolic environments such as drug treatments or nutrient deficiencies, which can be vastly useful in imitating HDAC inhibition by simulating the unique metabolic profile of HDAC inhibited cells and assessing the resulting metabolic shifts [59]. 

Shen et al. used constraint-based modeling (CBM) which simulates the GEM metabolic network according to constraints such as from gene expression data. This approach can be deployed to simulate different metabolic states, such as after HDAC inhibition [37]. CBM is capable of modeling the mechanistic relationships between genes, proteins, and metabolites, providing an extremely robust computational model of the metabolism of a cell. By utilizing CBM models derived from transcriptomics data, Shen et al. were able to discover a set of diverse novel observations, including novel interactions between histone acetylation/deacetylation and the enzymes citrate synthase and succinate dehydrogenase [37]. In addition to these novel metabolic interactions, Shen et al. discovered that cell lines with high acetylation fluxes were likely to be more sensitive to vorinostat (an HDACI). CBM presents itself as a promising approach which can be used in tandem with high-throughput cell screening methods to understand the complexities of cancer metabolism and HDAC inhibition. 

Another promising approach in the study of HDACIs is modeling of post-translational modifications acetylation and phosphorylation, both of which play large roles in regulating a variety of metabolic pathways. Smith et al. developed the comparative analysis of regulators of metabolism (CAROM) method, which can identify important features predictive of regulation by each post-translational modification using machine learning. CAROM uses metabolic fluxes, enzyme molecular weight, and catalytic activity, as well as topological properties of the pathway to determine putative regulatory sites of acetylation and phosphorylation. Using CAROM, Smith et al. predicted acetylation changes during the cell cycle. Upon comparison with the acetylation proteomics data from the Schölz et al. study described earlier, they were able to predict that growth inhibition caused by specific deacetylase inhibitors is likely to occur in the G2 phase of the cell cycle [60].

### 3.7. Microbiome Profiling

The role of the microbiome in regulating HDACs is yet to be fully determined, but there is strong evidence to suggest a link between microbiota, metabolism, and HDAC regulation. The microbiome represents a potentially untapped source of HDAC inhibitors, distinct from the current set of drugs available today. Bultman et al. discovered how dietary fiber can cause specific bacteria in the gut to produce short-chain fatty acids (SCFA) [61]. SCFAs are known to be key biomarkers of colorectal cancer and act as HDACIs [61]. Diets consisting of high dietary fiber lead to increase in microbiome-derived butyrate and propionate, both of which are HDAC inhibitors [61]. Donohoe et al. similarly observed the effects of butyrate on HDAC activity and the subsequent impact of colorectal cancer proliferation. Their results indicated that high levels of dietary fiber and butyrate were associated with lower HDAC levels and protection against colorectal cancer. This evidence suggests a key role that butyrate and other SCFAs play in regulating HDACs. So far, the identity of SCFA producing bacteria and their role in pathologies is still emerging. Yuille et al. screened a subset of commensal gut bacteria for HDAC inhibition. Their results indicate that *Megasphaera massiliensis* MRx0029 was correlated with an effective and specific inhibition of HDACs within the microbial community [62]. Researchers further characterized *M. massiliensis* MRx0029 as a butyrate and valeric acid producer, both of which are SCFAs with HDACI activity [62].

## 4. Conclusions

Abnormal epigenetic regulation, especially histone acetylation, underlies numerous pathologies. Inhibitors of histone deacetylases are a promising class of drugs that can reverse aberrant epigenetic changes in these diseases. Since both histone acetylation and deacetylation are highly sensitive to changes in the levels of numerous metabolites, metabolic activity can influence the effectiveness of histone deacetylase inhibitors. So far, our understanding of the interactions between metabolism, histone acetylation, and HDAC inhibitors remains incomplete.

Understanding the interdependencies between these processes is challenging due to the highly interconnected nature of both cellular metabolism and gene regulation (Figure 1). Here, we described how new systems technologies such as proteomics and metabolomics are revealing new interactions between these processes. Computational models complement these technologies and can allow scientists to integrate vast amounts of data and gain a better understanding of interaction mechanisms. Another challenge in uncovering these interactions is that most studies have focused on one data modality, such as transcriptomics or metabolomics alone. Multi-omic characterization of this interplay in a single biological system combined with innovations in machine learning and modeling can help bridge diverse data types and uncover hidden interactions [63].

Furthermore, it is likely that only a small fraction of the interactions between these central cellular processes have been characterized. Recently, a new interaction between the key redox metabolite NADPH and HDAC3 was discovered in adipocytes [64]. Novel high throughput functional genomics screening methods have identified HDAC6 as a regulator of glycolysis [65]. It is likely that there are several other metabolites like NADPH that may moonlight and play an epigenetic role. Recent innovations in measuring cellular compartment-specific metabolism and high-throughput measurement of protein–metabolite interactions may tease out how metabolites modulate HDACs [66,67].

While the roles of metabolism and HDACs are well documented in cancers, several studies have also uncovered a pivotal role for HDACs in glucose homeostasis [68], suggesting that HDAC inhibitors may be effective at mitigating diabetes and metabolic disorders. HDAC inhibitors are also being explored for treating immunological and neurological disorders. However, the heterogeneity in expression of HDACs makes it challenging to determine cases where HDAC inhibitors will be effective [69]. Leveraging metabolic assays and in vivo metabolic imaging methods as companion diagnostics can lead to the identification of patients who will benefit the most from HDAC inhibitors. Similarly, identifying synergistic combinations of metabolic inhibitors with HDAC inhibitors can enhance the efficacy of current therapies. In sum, combining cutting-edge systems biology tools including imaging, mass spectrometry, and artificial intelligence can uncover novel interactions between metabolism and epigenetic gene regulation, and lead to new therapies for numerous pathologies.

## Figures and Tables

**Table 1:** Table summary of common HDACIs’ isoform selectivity, structure, and connection to metabolism.

HDACI	HDAC Isoform Selectivity	Notable Structural Characteristics	Metabolic Relationship	Sources
Trapoxin (TPX)	HDAC1,4	Cyclic tetrapeptide Epoxyketone group	Activity decreased by reductive metabolism	[2,30]
Depsipeptide/FK228 (Romidepsin)	HDAC1,2 HDAC4, 6 (weaker)	Bicyclic peptide Activated by disulfide bond reduction	Activity increased by reductive metabolism Decreases glycolysis by suppressing c-Myc (glycolysis regulator)	[2,31,32,33]
Butyrate	HDAC1,2,3,4,5,7,8,9 (Class I, IIa)	Short-chain fatty acid anion (deprotonated carboxyl group)	Produced by gastrointestinal metabolism of fiber	[1]
Sodium Butyrate (NaB)	HDAC1,2,3,4,5,7,8,9 (Class I, IIa)	Short-chain fatty acid salt	Increases aerobic and mitochondrial metabolism	[1,19]
Trichostatin A (TSA)	HDAC1,3,4,6,10	Hydroxamic acid	Increases aerobic and mitochondrial metabolism	[19,26]
Valproate (VPA)	HDAC1,2,3,4,5,7,8,9 (Class I, IIa)	Short-chain fatty acid	Decreases glycolysis and lipid metabolism	[3]
Vorinostat/Suberoylanilide Hydroxamic Acid (SAHA)	HDAC1,2,3,4,5,6,7,8,9,10,11 (Class I, II, IV)	Hydroxamic acid	Decreases glycolysis	[2]
Panobinostat (LBH-589)	HDAC1,2,3,4,5,6,7,8,9,10,11 (Class I, II, IV)	Hydroxamic acid	Decreases glycolysis by suppressing c-Myc (glycolysis regulator)	[31,34]

**Table 2:** Table summary of usage, advantages, and disadvantages of relevant technologies for HDAC studies.

Technology	Primary Usage	Advantages	Disadvantages
Chromatin immunoprecipitation sequencing (ChIP-seq)	Epigenomics	Quantifies histone and other DNA-binding protein’s location on genome	High cost; reliance on highly sensitive and selective antibody
Single cell RNA sequencing (scRNA-seq)	Transcriptomics	Measures differentially expressed genes (e.g., response to HDACIs) in a variety of cell types	Poor cell quality control can lead to low-resolution results and inconsistent transcript coverage
Liquid chromatography-mass spectrometry (LC-MS)	Proteomics	Identifies residues of interest in post-translational modifications	High cost, sensitive to noise and it is not genome-scale unlike transcriptomics
^13^C Magnetic Resonance Spectroscopic Imaging (^13^C-MRSI)	Metabolomics	Selectable, tracer metabolites of interest; minimally invasive in vivo	Expensive to achieve the resolution required for measuring metabolic shifts

## Abbreviations

BS-seq	Bisulfite Sequencing
CAROM	Comparative Analysis of Regulators of Metabolism
CBM	Constraint-Based Modeling
CCLE	Cancer Cell Line Encyclopedia
CE-MS	Capillary Electrophoresis Mass Spectrometry
ChIP-seq	Chromatin Immunoprecipitation Sequencing
CTRP	Cancer Therapeutics Response Portal
ESC	Embryonic Stem Cell
FACS	Fluorescence-Activated Cell Sorting
FBP1	Fructose-1,6-bisphosphate
FK228	Depsipeptide (active form is Romidepsin)
GC-MS	Gas Chromatography Mass Spectrometry
GCP	Global Chromatin Profiling
GEM	Genome-Scale Metabolic Model
GLUT1	Glucose Transporter Type 1
HAT	Histone Acetyltransferase
HCC	Hepatocellular Carcinoma
HDAC	Histone Deacetylase
HXK1	Hexokinase 1
LC-MS	Liquid Chromatography Mass Spectrometry
MNase	Micrococcal Nuclease
MRSI	Magnetic Resonance Spectroscopic Imaging
MS	Mass Spectrometry
NaB	Sodium Butyrate
NMR	Nuclear Magnetic Resonance
NPC	Neuronal Precursor Cell
PBAT	Post-Bisulfite Adapter-Tagging
PPP	Pentose Phosphate Pathway
RNA-seq	RNA Sequencing
SAHA	Suberoylanilide Hydroxamic Acid (Vorinostat)
SCFA	Short-Chain Fatty Acids
scRNA-seq	Single-Cell RNA Sequencing
scRRBS	Single-Cell Reduced-Representation Bisulfite Sequencing
SFC-MS	Supercritical Fluid Chromatography Mass Spectrometry
SILAC	Stable Isotope Labeling by Amino Acids in Cell Culture
TPX	Trapoxin
TSA	Trichostatin A
VPA	Valproate
